# Community Health Worker-Delivered Mental Health Interventions for Latine Populations in the U.S.: A Systematic Literature Review

**DOI:** 10.1007/s10488-025-01459-6

**Published:** 2025-07-21

**Authors:** Erika L. Gustafson, Jacqueline O. Moses, Eliana Pimentel, Davielle Lakind, Viviana Uribe, Dillon Thorpe, Gabriella Bobadilla, Lily Caglianone, Grayson J. Dickinson, Dale Smith, Jennifer Westrick, Lisa Sánchez-Johnsen

**Affiliations:** 1 Institute for Juvenile Research, Department of Psychiatry, College of Medicine, University of Illinois Chicago, Chicago, USA; 2 Department of Clinical Psychology, College of Health Professions, Mercer University, Macon, USA; 3 Department of Psychology, Northern Illinois University, DeKalb, United States; 4 Department of Psychology, University of Illinois Chicago, Chicago, USA; 5 Psychology Department, College of Science and Health, DePaul University, Chicago, USA; 6 College of Osteopathic Medicine, William Carey University, Hattiesburg, USA; 7 Rush University Medical Center, Chicago, USA; 8 Institute for Health and Humanity, Department of Psychiatry and Behavioral Medicine, Medical College of Wisconsin, Milwaukee, USA; 9 University of Hawaii at Manoa, Honolulu, USA

**Keywords:** Community health workers, Promotores de salud, Mental health, Psychological treatment, Latine/x/o/a, Systematic review

## Abstract

The United States (US) faces a mental health crisis characterized by persistent unmet mental health needs, provider shortages, and pronounced mental health inequities for systematically marginalized communities, including the Latine population. Integrating community health workers (CHWs) into mental healthcare delivery via task-shifting is one promising approach to address longstanding inequities in treatment access. Yet, most studies of CHW mental health models have been conducted outside of the US, necessitating an evaluation of the evidence base for these models domestically, and in particular, their use with Latines living in the US. This systematic review examines the evidence for CHW-delivered mental health interventions for US-based Latines. In total, 27 articles (25 trials) met the eligibility criteria. The majority of interventions were delivered in Spanish to immigrant populations. The most common mental health targets included depression, stress, and parenting or the parent–child relationship. Common intervention components included psychoeducation, general coping skills, behavior management, case management, communication skills, relaxation, and problem solving. The majority of studies found that CHW-delivered interventions led to significant mental health symptom improvements. However, there was a high risk of bias across studies. Thus, there is promising preliminary evidence supporting CHW-delivered mental health interventions for US Latines, but more rigorous evaluation of these models is needed.

**Supplementary Information:**

The online version contains supplementary material available at 10.1007/s10488-025-01459-6.

## Introduction

### Unmet Mental Health Needs Among Latines

Significant unmet mental health needs in the United States (US) are well documented—50% of adults and youth with mental health needs do not receive mental healthcare (National Institute of Mental Health, 2023; Whitney & Peterson, 2019). These unmet needs are more pronounced for communities systematically excluded in standard models of care, including Latines^1^ Latine adults and youth are approximately half as likely to receive mental health services compared to their non-Latine counterparts (Marrast et al., 2016; Substance Abuse & Mental Health Services Administration, 2021). Additionally, when Latine people do access services, they are less likely to receive guideline-congruent (i.e., evidence-based) care (Cabassa et al., 2006). Latines make up approximately 20% of the US population, are the second fastest growing ethnic group, and constitute a large portion of the US’ immigrant population which is projected to be the primary driver of the country’s population growth (Krogstad et al., 2023; Moslimani & Passel, 2024; Vespa et al., 2020). Hence, mental health disparities in this population pose a public health imperative, necessitating improvements and innovations to our mental health service models.

The magnitude of mental health needs far outpaces the US’ mental health workforce capacity, and this provider shortage is a major contributor to the mental health treatment gap. Approximately one in five counties have insufficient non-prescribing providers (i.e., psychologists, counselors, social workers) to meet existing mental health needs, with rural and low-income communities facing greater shortages (Butryn et al., 2017). The scarcity of providers is even more pronounced when considering cultural and linguistic competency factors. Only 6% of psychologists, 11% of counselors, and 14% of social workers are Latine, and between 2014–2019, the proportion of U.S. facilities offering Spanish services declined by 17.8% (American Counseling Association, 2024; American Psychological Association, 2022; National Association of Social Workers, 2020; Pro et al., 2022). The shortage of linguistically and culturally appropriate services poses a significant barrier for Latine families, the majority of whom speak Spanish (Krogstad et al., 2023; Lopez et al., 2018), and have a strong preference for bilingual providers over the use of interpreters with an English-speaking provider (Villalobos et al., 2016).

### The Potential of Community Health Workers in Mental Health

Integrating community health workers (CHWs) into mental healthcare delivery via task-shifting is one promising approach to address longstanding inequities in treatment access. CHWs are trusted members of the community served, and as such, have a close understanding of and experience with the community, including shared language and culture; they act as a link between the community and health services to facilitate access and engagement (American Public Health Association, 2016). Task-shifting involves training non-specialists, i.e., those without advanced mental health training or formal degrees such as CHWs, to deliver psychological supports, including brief, low-intensity interventions (Raviola et al., 2019). CHWs in a mental health interventionist role present an avenue through which to increase access to care by mitigating barriers related to the general provider shortage as well as the shortage of providers able to work with linguistically and culturally diverse populations (Barnett et al., 2018a, 2018b).

CHW mental health models are particularly relevant for the Latine community. Latine people show a strong preference for initially seeking help through informal sources of support, such as family, friends, and community leaders (Gearing et al., 2024). Several factors may contribute to this pattern of help seeking behavior. Seeking support from family, friends, and community members aligns with the cultural values of *familismo* (i.e., the importance of the family unit, and emphasis placed on family relationships, supports, and responsibilities) and *personalismo* (i.e., valuing interpersonal relationships characterized by warm and caring interactions; Calzada et al., 2013; Davis et al., 2019). Additionally, given the legacy of systemic racism in US healthcare (Feagin & Bennefield, 2014), there is a valid mistrust of the medical system that may further incline Latine people to first seek informal sources of support within their network before seeking professional help. Given CHWs’ positionality as “near-peers” and “boundary spanners” (i.e., members of the community who are also connected to formal health systems), incorporating this workforce into mental healthcare delivery better aligns service models with the help seeking practices seen in the Latine community while also bringing more community members into the spectrum of care. Further, research has shown that clients’ sense of providers’ *cultural understanding* or *competence* is associated with improved client outcomes in mental health services (Chu et al., 2023; Soto et al., 2018). Since the CHW role is characterized by having a deep understanding of the community served, this workforce is particularly poised to facilitate clients’ sense of cultural understanding, and by extension, promote improved outcomes in mental health interventions.

There is a growing evidence base demonstrating that CHWs can effectively deliver mental health interventions that lead to significant patient symptom reduction (Barnett et al., 2018a, 2018b; Bunn et al., 2021; Singla et al., 2017; Van Ginneken et al., 2013). In their review of 27 trials, Singla et al. (2017) found moderate effect sizes for CHW-delivered treatments for common mental health disorders across low- and middle-income countries (LMIC). Barnett et al. (2018a, 2018b) review of 39 trials included CHW-delivered mental health treatments in both the US and LMIC and found that most of these trials led to significant symptom reduction. Yet, a limitation in the current literature is that the majority of research on CHW-delivered mental health interventions has been conducted in LMIC; trials in LMIC were twice as common as US-based studies (26 trials in LMIC vs 13 trials in the US; Barnett et al., 2018a, 2018b). Task-shifting CHW models emerged in response to the limited formal mental health workforce in LMICs, so it follows that the majority of literature to date reflects research conducted outside of the US (Bunn et al., 2021; Le et al., 2022; Singla et al., 2017). However, high-income countries, including the US, also face significant mental health provider shortages that limit their ability to meet population wide mental health needs. Juxtaposing the US’ large and growing Latine population (Krogstad et al., 2023) facing persistent mental health inequities against the limitations of the mental health workforce, it becomes evident that the US also stands to benefit from integrating CHWs into the spectrum of mental healthcare to address unmet mental health needs in the Latine community.

### Present Study

If CHW mental health models are to be implemented at scale and with the Latine community in the US, it is necessary to understand the use of these models domestically. To date, one systematic review has focused on CHW mental health models in the US (Weaver & Lapidos, 2018); however, it reviewed studies through 2016 and did not have a specific focal population. Therefore, the present systematic review aimed to provide an updated review of the literature on CHW mental health services in the US, with a focus on these services within the Latine community. This review was guided by the following questions: (1) What is the current state of the evidence for CHW-delivered mental health interventions for US-based Latine populations? (2) What cultural and contextual factors, if any, are present in CHW mental health interventions for this population?

## Methods

### Search Strategy

A comprehensive literature search was developed collaboratively by the lead author (ELG) and an experienced medical librarian (JW). The literature search was conducted by JW in June 2022 in the following databases: PubMed/MEDLINE, Scopus, CINAHL, and PsycINFO. Google Scholar was searched as well. Both controlled vocabularies (e.g. MeSH terms) and keywords in the title or abstract fields were searched. There were no restrictions on the age of participants or language of publication. Geographically, articles were restricted to US-based interventions. Additionally, a hand search was conducted of the reference lists of selected articles. A reproducible search strategy is attached—see Appendix. This review followed the Preferred Reporting items for Systematic Reviews and meta-Analyses (PRISMA) guidelines (Page et al., 2021), and was registered in PROSPERO (registration ID CRD42022339619), the international prospective register of systematic reviews, prior to conducting the literature search.

### Inclusion & Exclusion Criteria

For inclusion, studies had to meet criteria related to (1) workforce role, (2) intervention focus, (3) target population, (4) outcomes, and (5) study design. (1) The *workforce* implementing the intervention had to be composed of CHWs. The CHW role can go by many names (e.g., promotora de salud, paraprofessional, lay health provider, etc.), which were included in the key search terms. Therefore, the workforce criterion was evaluated based on alignment with the definition of the role rather than a specific title. We used the American Public Health Association’s (APHA) definition of CHWs as “a frontline public health worker who is a trusted member of and/or has an unusually close understanding of the community served” (American Public Health Association, 2016). Additionally, the CHWs had to serve in an interventionist role (i.e., delivering mental health intervention content). Studies where CHWs only provided navigation or case management were excluded. This was to ensure that our review could speak to CHWs’ ability to implement psychological interventions. (2) The *intervention* had to include psychological content targeting a mental health issue (e.g., depression, anxiety, stress, etc.). Studies in which the intervention only targeted a physical health issue were excluded; however, if the intervention had dual physical and mental health targets, then they were included. (3) The *target population* had to be comprised of Latine people in the U.S. The sample had to be either majority Latine, or if Latines comprised less than 50% of the sample, the study had to include subsample analyses for the Latine subgroup. This was to ensure that our review could speak to the effects of these interventions specifically for U.S.-based Latines. (4) *Outcomes* had to include patient-level mental health symptom measures. If available, implementation outcomes (e.g., fidelity, satisfaction, participation) were coded as a secondary outcome, but studies only focused on implementation without mental health outcomes were excluded. (5) *Study designs* included randomized control trials (RCTs), quasi-experimental, pre/post, and mixed methods studies. We excluded case reports, meta-analyses, and study protocols. Articles had to be published in a peer-reviewed journal (i.e., book chapters, dissertations, and conference abstracts were excluded).

### Study Selection

Results from the literature search were uploaded into Covidence, an online screening and data extraction tool for conducting systematic reviews. Article titles and abstracts were independently screened by two members of the review team (composed of authors ELG, JOM, EP, DL, and VU), evaluating them against the inclusion criteria. Articles that made it through the abstract screening stage then had their full texts independently reviewed by two members of the review team. Regular team meetings were held to discuss questions and resolve discrepancies about inclusion via consensus.

### Data Extraction & Coding

The lead author (ELG) designed an online data extraction form using the Research Electronic Data Capture (REDCap) platform. Prior to beginning formal data extraction, this form was piloted with the review team to refine data extraction fields and definitions, as well as build consistency in data extraction. Then, pairs of coders from the review team independently reviewed and extracted data from each article. Regular consensus meetings were held with the review team to resolve discrepancies in data extraction coding.

### Quality Assessment

We used the JBI Critical Appraisal Tools for Assessment of Risk of Bias to evaluate study rigor (Munn et al., 2023). JBI has quality appraisal guides across different study types, which was appropriate for this review given the inclusion of articles with varying study designs. We used the following guides: the Critical Appraisal Tool for Assessment of Risk of Bias for RCTs, the Checklist for Quasi-Experimental Studies, and the Checklist for Qualitative Research (to evaluate the qualitative portion of mixed methods studies). The Checklist for Quasi-Experimental Studies was used for studies with either a quasi-experimental or a pre/post design, consistent with JBI instructions for this tool. Using the appraisal guide corresponding to an article’s respective study design, each article was coded independently by two reviewers from the quality assessment team (DT, LC, and GJD). The two exceptions to this coding approach were for appraisal items related to evaluating statistical analyses and qualitative analyses. For statistical items, each article was coded by a senior data scientist (DS) to ensure expert evaluation of statistical methods and interpretation. Members of the coding team with robust qualitative experience (ELG, DK, and JOM) completed the qualitative evaluations. Regular discussion meetings were held to resolve questions and discrepancies via consensus.

### Data Synthesis

The articles in this review were variable in design, methodologies, outcome measures and reporting; hence, a meta-analytic approach was not deemed appropriate. Instead, we followed standard guidelines for conducting narrative synthesis (Popay et al., 2006). Using textual descriptions, tabulation, and vote counting in a common rubric (i.e., the dataset generated from aggregating the data extraction forms for each study), we synthesized data across the following domains: study and sample characteristics (e.g., study design, sample size, sample descriptives such as age, ethnicity, etc.), CHW characteristics (e.g., descriptives such as age, ethnicity, etc., CHW education, training and supervision, etc.), intervention characteristics (e.g., mental health targets, intervention components, tailoring, etc.), and intervention outcomes (e.g., symptom change, implementation outcomes).

## Results

### Study Descriptives

**Fig. 1:**
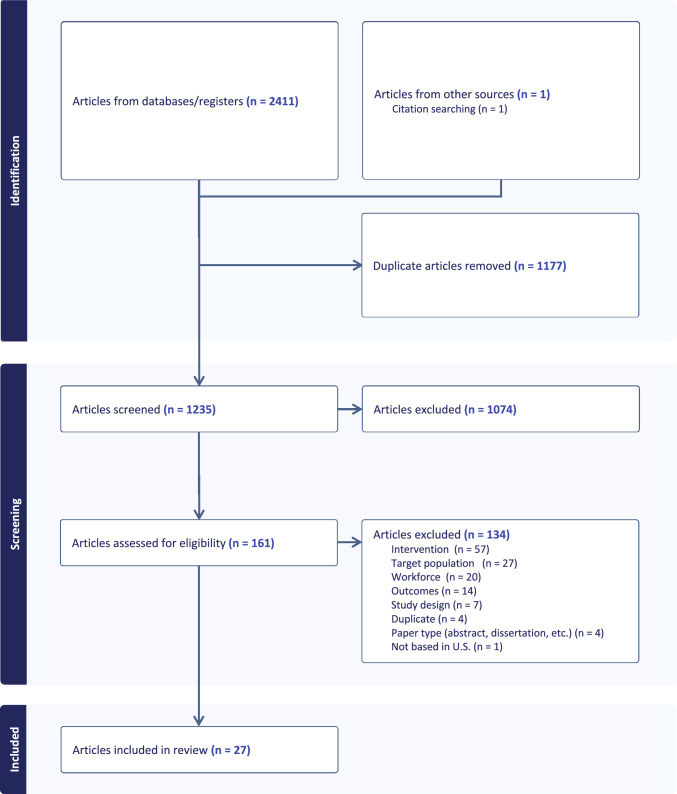
PRISMA flow diagram

After removing duplicates from Covidence, 1,235 titles and abstracts were independently screened in duplicate by two reviewers. One-hundred sixty-one full-text articles were assessed for eligibility, also in duplicate, resulting in 27 articles selected for inclusion. See Fig. 1 for the PRISMA flow diagram. The 27 articles represented 25 unique studies, as two studies were reported in multiple publications. Of these 25 studies, 14 studies (56%) were RCTs, and the remaining studies were pre-post or quasi-experimental designs. Mixed methods were used in five studies (20%). Included articles are summarized in Table 1.

**Table 1:** Study design & sample characteristics

Study	Study design	Client N (intervention condition)	Client age group	Client Latine background*	Client sample majority immigrant	CHW N	CHW Latine	CHW educational background	CHW experience
Alegría et al. (2019)	RCT	307 (153)	Older adult	Hispanic: unspecified, but one study site in Puerto Rico	Y	NR	NR	NR	NR
Ashing and Rosales (2014)	RCT	221 (110)	Adult	Latina-Americans: majority of Mexican descent; others unspecified	Y	2	NR	> 2 year college education	> 3 year experience working with underserved communities
Borelli et al. (2021)	Pre/post	112	Family: parent/child	Latinx: majority of parents born in Mexico; others unspecified; children unspecified	Y	5	Y	NR	Trained community workers drawn from, and respected by, the local residents
Camacho et al. (2015)	Pre/post	189	Adult	Hispanic/Latinos: Mexican & Mexican-Americans	NR	NR	Y	Physicians trained in Mexico but not licensed to practice medicine in the United States	Had background as research coordinators as well as knowledge in prevention and management of chronic diseases, but not in mental health treatment
Conway et al. (2004)	RCT	143 (71)	Family: parent/child	Latinos: majority of parents from Mexico; others unspecified; children unspecified	Y	NR	Y	NR	NR
Crisanti et al. (2019)	RCT	291 (146)	Adult	Hispanic: unspecified	NR	2	NR	Certified in the delivery of peer support services through a state-level credentialing board	In recovery from a substance use disorder. All group facilitators were from the study catchment area and therefore representative of the target population
Cutshaw et al. (2012)	Pre/post	305	Adult	Hispanic: unspecified, but study site in US-Mexico border community	Y	2	Y	NR	Had lived in the community 20 to 35 years. Some had prior experience facilitating the intervention during the pilot phase
Hernandez and Lucero (1996)	Pre/post	61 families	Family: parent/child	Hispanic: parents & children unspecified	N	NR	Y	NR	Grew up in similar inner-city Hispanic communities. The staff have also shown an ability to relate to families in the program and gain their trust and confidence
Hovey et al. (2014)	Pre/post	6	Adult	Latinas: all from Mexico	Y	1	Y	NR	Experince as migrant farmworker
Joachim-Célestin et al. (2022)	Quasi-experimental	98 (43)	Adult	Latinas: majority of Mexican descent; others unspecified	NR	2	NR	Certified CHWs	NR
Knox et al. (2011)	RCT, mixed methods	282 (140)	Family: parent/child	Latinos: majority of parents from Mexico; majority of children born in US, others unspecified	Y	NR	Y	NR	From the community served; experience with community programming
Lopez et al. (2019)	RCT	29 (14)	Family: parent/child	Latinx: majority of parents from Mexico; some from Guatemala, Cuba, El Salvador, Peru, or born in US; majority of children born in US, others unspecified	Y	6	Y	NR	Latinx mothers of children (8 and up) with autism, and leaders in their community
Magaña et al. (2015)	RCT	100 (50)	Adult	Latinas: majority of Mexican decent; others unspecified	Y	7	Y	NR	Latina mothers caring for a child with intellectual and developmental disabilities, and living in the same geographic area as participants
Magaña et al. (2017)	Pre/post, mixed methods	22	Family: parents	Latinos: majority of parents Mexican/Mexican American; some Puerto Rican; others unspecified	Y	3	Y	NR	Mothers of children with an ASD or developmental disability, and identified by CBO as having leadership qualities
Magaña et al. (2020)	RCT	109 (54)	Family: parent/child	Latinos: majority of parents of Mexican descent; some from Central America, Caribbean, & South America; children unspecified	Y	16	Y	NR	Promotoras were mothers with children with ASD and trained to be peer mentors
Moore et al. (2016)	RCT	29 (14)	Adult	Latinos: majority from Mexico; some from Central America, South America, or born in US	Y	4	Y	NR	Latina immigrants with 3-8yrs of experience as health promoters
Nicolaidis et al. (2013)	Pre/post, mixed methods	10	Adult	Latinas: majority from Mexico; other from Guatemala	Y	1	Y	NR	Mexican-born woman with several decades of experience serving as a domestic violence advocate and CHW
Norr et al. (2003)	RCT	588 (258)	Family: parents	Latinos: Mexican–American	NR	NR	Y	NR	CHWs had similar lived experinces as clients, such as in poverty, violence, teen pregnancy and stress. CHWs were recruited from already existing agencies that serviced the same neighborhoods
Rotheram-Fuller et al. (2017)	RCT	203 (99)	Family: parents	Latinas: unspecified	N	9	NR	NR	Parents with children in K-12th grade school; postive role models in the local communities served
Schepens Niemiec et al. (2018, 2021)	Pre/post, mixed methods	40	Older adult	Latinos: majority from Mexico; some from Central America or born in US; others unspecified	Y	2	NR	NR	NR
Schwingel et al. (2017)	Pre/post, mixed methods	34	Older adult	Latinas: majority of Mexican origin; some from Guatemala, Colombia, or born in US	Y	3	Y	At least high school education	Latina women who demonstrated a high degree of familiarity with the local Latino communityas defined by community organization leaders. Each had served the community organization as volunteers for many years and were respected members of the community
Spencer et al. (2013)	RCT	164 (72)	Adult	Latinos: majority of Mexican origin; others unspecified	NR	NR	Y	NR	NR
Tran et al. (2014)	Pre/post	58	Adult	Latinas: majority from Mexico; some from South America and Caribbean/Central America	Y	48	Y	NR	NR
Wagner et al. (2015, 2016)	RCT	107 (61)	Adult	Latinos: majority Puerto Rican; others unspecified	N	1	Y	High school diploma and Certified Nursing Assistant	2 years of experience caring for patients with diabetes. Experience leading religious education groups for several years, so was connected to and respected by the Latino church community
Williamson et al. (2014)	RCT	194 (113)	Family: parent/child	Latinas: majority of mothers born in Mexico; others unspecified; majority of children born in US, others unspecified	Y	NR	Y	NR	NR

*Latine background descriptions mirror the language used in each respective article. Hence the variable use of Hispanic, Latino/a/x, etc.

### Client/Participant Sample Characteristics

Across all studies, sample size ranged from six to 588 clients/participants (*M* = 48, *SD* = 133). Client samples across the twenty-five studies fell into three age groups: adult, older adult, and family (parent/child or parent focused). Twelve studies (48%) reported samples of adult participants. Three studies (12%) focused on older adult samples, defined as studies in which the authors specified targeting an “older adult” population and/or specified inclusion criteria of age 60 or above. The remaining 10 studies (40%) reported samples of parents or parent–child dyad participants. Per our inclusion criteria, all study samples included Latine clients. Twenty studies (80%) provided data on specific Latine background among client samples. The most frequently reported Latine background was Mexican. Less commonly reported Latine backgrounds included Central American (e.g., Salvadorian, Guatemalan), South American (e.g., Peruvian, Colombian), and Caribbean (e.g., Puerto Rican, Cuban). Seventeen studies (68%) specified a majority immigrant client sample.

### CHW Sample Characteristics

Seventeen studies (68%) reported the number of CHWs involved in the delivery of interventions. The number of CHWs ranged from 1 to 48 (*M* = 6.71, *SD* = 11.30), with 114 CHWs in total across studies. Studies used a variety of terms to refer to CHW providers, with the most common title being promotora/es. Other titles included paraprofessional interventionist, mentor mother, depression care specialist, health advocate, and peer provider. Sixteen studies (64%) reported the gender of the CHW providers; of those, 15 studies had exclusively female CHWs, and one study had one male and one female CHW. Most studies (80%) did not report CHW race or ethnicity. However, given that the title of “promotor/a” is predominantly used for Latine CHWs, we used this title as a proxy denoting Latine ethnicity. With this coding scheme, 19 studies (76%) had Latine CHW providers. Of these, four studies reported the specific Latine background of the CHW provider, which included Mexican and Puerto Rican. With regard to the CHW role in interventions, 20 studies (80%) had CHWs as the sole provider, in four studies (16%) they were a co-provider, and one study (4%) did not specify.

Regarding CHWs’ education background, six studies (24%) reported educational history. Educational backgrounds varied, ranging from at least a high school education or diploma (Schwingel et al., 2017), certification as a CHW (Joachim-Célestin et al., 2022) or peer support specialist (Crisanti et al., 2019), to a certified nursing assistant (Wagner et al., 2015, 2016), and physicians trained in Mexico but not licensed to practice medicine in the US (Camacho et al., 2015). While formal education was rarely reported, background experience was reported in 19 studies (76%). Background experiences included prior experience working with the community served, or being from the community served (e.g., in recovery from a substance use disorder, mothers of children with autism spectrum disorder, having lived in the target neighborhood for decades, etc.). Most reported background experiences aligned with the construct of social proximity. Previously described in MASKED, social proximity refers to a closeness to the community served, either through community membership or through intimate knowledge and understanding of the community. Many of the background experiences of CHWs mapped onto the construct of social proximity via experiences of shared ethnicity, community, or lived experience with a mental health disorder. Using this definition, 21 studies (84%) had CHWs who reflected social proximity in relation to their population served.

### CHW Training & Supervision

Reports of CHW training and supervision activities are summarized in Table 2 and described below.

**Table 2:** Training & supervision activities reporting for CHW-delivered mental health interventions

Study	Training activities	Training provider	Supervision/support activities	Supervision/support provider
Alegría et al. (2019)	Y	Y	Y	Y
Ashing and Rosales (2014)	Y	Y	Y	Y
Borelli et al. (2021)	Y	Y	NR	NR
Camacho et al. (2015)	Y	Y	NR	NR
Conway et al. (2004)	NR	NR	NR	NR
Crisanti et al. (2019)	Y	NR	NR	NR
Cutshaw et al. (2012)	Y	NR	NR	NR
Hernandez and Lucero (1996)	Y	NR	NR	NR
Hovey et al. (2014)	Y	Y	NR	Y
Joachim-Célestin et al. (2022)	Y	NR	NR	NR
Knox et al. (2011)	Y	Y	Y	Y
Lopez et al. (2019)	Y	Y	Y	Y
Magaña et al. (2015)	Y	Y	Y	Y
Magaña et al. (2017)	Y	NR	NR	NR
Magaña et al. (2020)	Y	Y	Y	Y
Moore et al. (2016)	Y	Y	Y	Y
Nicolaidis et al. (2013)	Y	Y	Y	Y
Norr et al. (2003)	Y	Y	Y	Y
Rotheram-Fuller et al. (2017)	Y	Y	Y	Y
Schepens Niemiec et al., (2018, 2021)	Y	Y	Y	Y
Schwingel et al. (2017)	Y	Y	NR	NR
Spencer et al. (2013)	Y	NR	NR	NR
Tran et al. (2014)	Y	Y	Y	Y
Wagner et al. (2015, 2016)	Y	Y	Y	Y
Williamson et al. (2014)	Y	Y	Y	Y

#### Training

Twenty-four (96%) studies provided some report of training content for CHWs, and 18 studies (72%) reported the provider of the training. Trainers included study team members (e.g., Ashing & Rosales, 2014), programs leaders (e.g., Magaña et al., 2020), and clinical providers (e.g., Camacho et al., 2015). The duration of training was highly variable, ranging from 18 total hours over nine training sessions (Schwingel et al., 2017), to ongoing training over the course of 6 months (Norr et al., 2003). Training content across studies included education on specific conditions, such as physical and mental health issues (e.g., Hovey et al., 2014; Nicolaidis et al., 2013; Spencer et al., 2013), and education on specific intervention content and delivery, (e.g., Ashing & Rosales, 2013; Camacho et al., 2015; Hernandez & Lucero, 1996). Several studies reported using roleplays to support CHWs in their learning (e.g., Alegría et al., 2019; Magaña et al., 2017; Moore et al., 2016).

#### Supervision

Of the 25 studies, 15 (60%) reported the supervision provider, and 13 (52%) described the supervision process. Descriptions of supervision activities varied greatly. One detailed account described the role of the PI and study coordinator in offering biweekly supervision and training, focusing on broad study issues, ethical and cultural concerns, and staff support, adopting a clinical psychology supervision approach (Ashing & Rosales, 2014). Other supervision activities included the use of fidelity checklists (Alegría et al., 2019; Lopez et al., 2019), review of recorded sessions (Alegría et al., 2019; Ashing & Rosales, 2014), debriefing sessions to discuss cases and problem-solve (Knox et al., 2011; Lopez et al., 2019; Magaña et al., 2015), and role plays (Moore et al., 2016). Overall, the level of detail provided to describe supervision activities was limited, pointing to a need for more comprehensive documentation of supervision practices.

### Intervention Characteristics

Intervention characteristics are summarized in Table 3. Twenty-four studies (96%) specified the settings in which interventions were delivered. The majority of studies (64%) had interventions delivered in a single setting, while the remaining studies had interventions delivered across two or three settings. Thirteen studies included home visit based interventions; 12 studies included community-based interventions (e.g., churches, community-based social service agencies); seven studies included phone or telehealth-based interventions; two studies included school-based interventions; one study included a healthcare setting; and one study included a mental healthcare setting.

**Table 3:** Characteristics of CHW-delivered interventions

Study	CHW role	Intervention setting	Intervention language	Intervention focus	Intervention evidence level	Mental health targets	Mental health intervention components
Alegría et al. (2019)	Sole provider	Community-based, home visits, phone/telehealth	English, Spanish, Cantonese or Mandarin	Mental + physical health	Evidence Informed	Depression, anxiety, other	Psychoeducation, relaxation, behavioral activation, cognitive therapy, communication, CM/navigation
Ashing and Rosales (2014)	Sole provider	Phone/telehealth	English & Spanish	Mental health-only	Evidence Informed	Depression, stress	Psychoeducation, general coping skills, problem solving,CM/service navigation
Borelli et al. (2021)	Sole provider	Community-based	English & Spanish	Mental health-only	Evidence Informed	Parenting/parent–child relationship, other	Psychoeducation, other
Camacho et al. (2015)	Sole provider	Healthcare setting	Spanish	Mental health-only	EBT	Depression	Problem solving
Conway et al. (2004)	Sole provider	Home visits, phone/telehealth	Spanish	Mental health-only	Evidence Informed	Substance use (tobacco)	Behavior management, problem solving, other
Crisanti et al. (2019)	Sole provider	Mental health setting	NR	Mental health-only	EBT	Trauma, substance use (unspecified)	Psychoeducation, relaxation, general coping skills, other
Cutshaw et al. (2012)	Sole provider	Community-based	English & Spanish	Mental + physical health	Evidence-informed	Depression	Psychoeducation, behavioral activation, general coping skills
Hernandez and Lucero (1996)	NR	Home visits, school	English & Spanish	Mental health-only	Novel	Parenting/parent–child relationship, substance use (alcohol & other drugs), other	Psychoeducation, general coping skills, communication skills, communication, problem solving, CM/service navigation
Hovey et al. (2014)	Co-provider	Community-based	Spanish	Mental health-only	Evidence Informed	Depression, anxiety, stress, other	Psychoeducation, behavioral activation, exposure, cognitive therapy, general coping skills, problem solving, other
Joachim-Célestin et al. (2022)	Sole provider	Community-based, school	Spanish	Mental + physical health	Evidence Informed	Stress	General coping skills, CM/service navigation
Knox et al. (2011)	Sole provider	Community-based, home visits	NR	Mental health-only	EBT	Parenting/parent–child relationship, behavioral dysregulation, other	Psychoeducation, behavior management, communication skills, other
Lopez et al. (2019)	Sole provider	Home visits	English & Spanish	Mental health-only	Evidence Informed	Depression, stress, parenting/parent–child relationship, autism	Psychoeducation, behavior management, general coping skills, communication skills
Magaña et al. (2015)	Sole provider	Home visits	Spanish	Mental + physical health	Evidence Informed	Depression, stress	Psychoeducation, relaxation, other
Magaña et al. (2017)	Sole provider	Home visits	Spanish	Mental health-only	Evidence Informed	Depression, stress, parenting/parent–child relationship, autism	Psychoeducation, behavior management, general coping skills, communication skills, CM/service navigation
Magaña et al. (2020)	Sole provider	Home visits	English & Spanish	Mental health-only	Evidence Informed	Parenting/parent–child relationship, autism, other	Psychoeducation, behavior mangament, general coping skills, communication skills
Moore et al. (2016)	Sole provider	Community-based	Spanish	Mental health-only	Evidence Informed	Substance use (alcohol)	Psychoeducation, MI, CM/service navigation
Nicolaidis et al. (2013)	Co-provider	Community-based	Spanish	Mental health-only	Evidence Informed	Depression, stress, other	Psychoeducation, MI, cognitive therapy, CM/service navigation
Norr et al. (2003)	Co-provider	Home vists, phone/telehealth	English & Spanish	Mental + physical health	Evidence Informed	Parenting/parent–child relationship	Psychoeducation, behavior management, CM/service navigation
Rotheram-Fuller et al. (2017)	Sole provider	Home visits, phone/telehealth	English, Spanish, & Korean	Mental + physical health	Evidence Informed	Depression, other	Psychoeducation, relaxation, behavior management, general coping skills, problem solving, other
Schepens Niemiec et al. (2018, 2021)	Co-provider	Community-based, home visits, phone/telehealth	Spanish	Mental + physical health	Evidence Informed	Stress, other	MI, general coping skills, CM/service navigation
Schwingel et al. (2017)	Sole provider	Community-based	Spanish	Mental + physical health	Evidence Informed	Stress	Psychoeducation, relaxation
Spencer et al. (2013)	Sole provider	Community-based, home visits, phone/telehealth	English & Spanish	Mental + physical health	Evidence Informed	Stress	MI, general coping skills, CM/service navigation
Tran et al. (2014)	Sole provider	NR	Spanish	Mental health-only	Novel	Depression, stress	Psychoeducation, relaxation, general coping skills, other
Wagner et al. (2015, 2016)	Sole provider	Community-based	English & Spanish	Mental + physical health	Evidence Informed	Depression, anxiety, stress	Psychoeducation, relaxation, cognitive therapy, communication skills, other
Williamson et al. (2014)	Sole provider	Home Visits	Spanish	Mental health-only	Evidence Informed	Parenting/parent–child relationship	Psychoeducation, behavior management, communication skills, CM/service navigation, other

CM: case management

Twenty-three studies (92%) reported the language used to deliver the interventions. All of these 23 studies reported intervention delivery in Spanish, with 10 studies reporting delivery in Spanish and additional languages, including English, Cantonese, Mandarin, and Korean.

Ten studies (40%) included both mental and physical health targets (e.g., physical activity, healthy diet, diabetes), while 15 (60%) included only mental health targets. Most studies (72%) had more than one mental health target. Twelve studies addressed depression; twelve addressed stress; eight addressed parenting or the parent–child relationship; four addressed substance use; three addressed anxiety; three addressed autism spectrum disorder (ASD); one addressed trauma; one addressed behavioral dysregulation; and nine studies included an intervention target characterized as “other,” which referred to targets that did not correspond to a specific disorder or mental health challenge, but were related to mental health. These “other” targets included general mental wellbeing, social support, self-efficacy/empowerment, and self-esteem, amongst others.

Regarding intervention content, interventions consisted of multiple intervention components, ranging from one to seven components (*M* = 3.76, *SD* = 1.36). Twenty studies included psychoeducation; 13 included general coping skills; eight included behavior management; eight included case management/service navigation; eight included communication skills; seven included relaxation; seven included problem solving; four included motivational interviewing (MI); four included cognitive therapy; three included behavioral activation; and one included exposure. Ten studies also contained intervention components that did not fit within the categories above, and thus were coded as “other.” These intervention components included enhancing social support, goal-setting, healthy peer and romantic relationships, safety planning for domestic violence, increasing hopefulness and self-esteem, and assertiveness training, amongst others. In accordance with Barnett et al.’s (2018a, 2018b) definitions, three studies (12%) delivered EBTs; 20 studies (80%) delivered evidence-informed interventions, and two studies (8%) delivered novel interventions. Of note, we encountered ambiguity around what authors “counted” as a mental health intervention. For example, some studies conceptualized stress reduction as a mental health intervention, while others described a focus on stress reduction but did not frame this as a mental health intervention. Within our review, we conceptualized stress management-focused interventions as mental health interventions.

### Intervention Tailoring

Twenty-four studies (96%) reported conducting some type of cultural tailoring to address relevancy for Latine participants. Tailoring approaches included the incorporation of culturally relevant values, topics or content, and delivery techniques. Some studies described incorporating Latine values into interventions, such as *simpatia*, *respeto*, *personalismo*, and *familismo* (Hovey et al., 2014; Moore et al., 2016), or through *dichos*—common Spanish sayings or metaphors that reflect values (Magaña et al., 2017). Moore et al. (2016) expanded intervention content to address social stressors relevant to the immigrant context by including topics of acculturation stress, discrimination and poverty. Another study incorporated the roles of family and faith into the intervention curriculum to align with the cultural priorities of older Latinas (Schwingel et al., 2017). Studies also tailored the way in which intervention content was delivered, such as using *cuentos* or storytelling to communicate intervention content through a culturally relevant medium (Hernandez & Lucero, 1996; Magaña et al., 2015), or using lay-friendly language and familiar, safe settings (e.g. churches, schools, community-based organizations) to frame the intervention as non-threatening (Alegría et al., 2019; Hovey et al., 2014; Joachim-Célestin et al., 2022; Knox et al., 2011).

### Outcomes

**Fig. 2:**
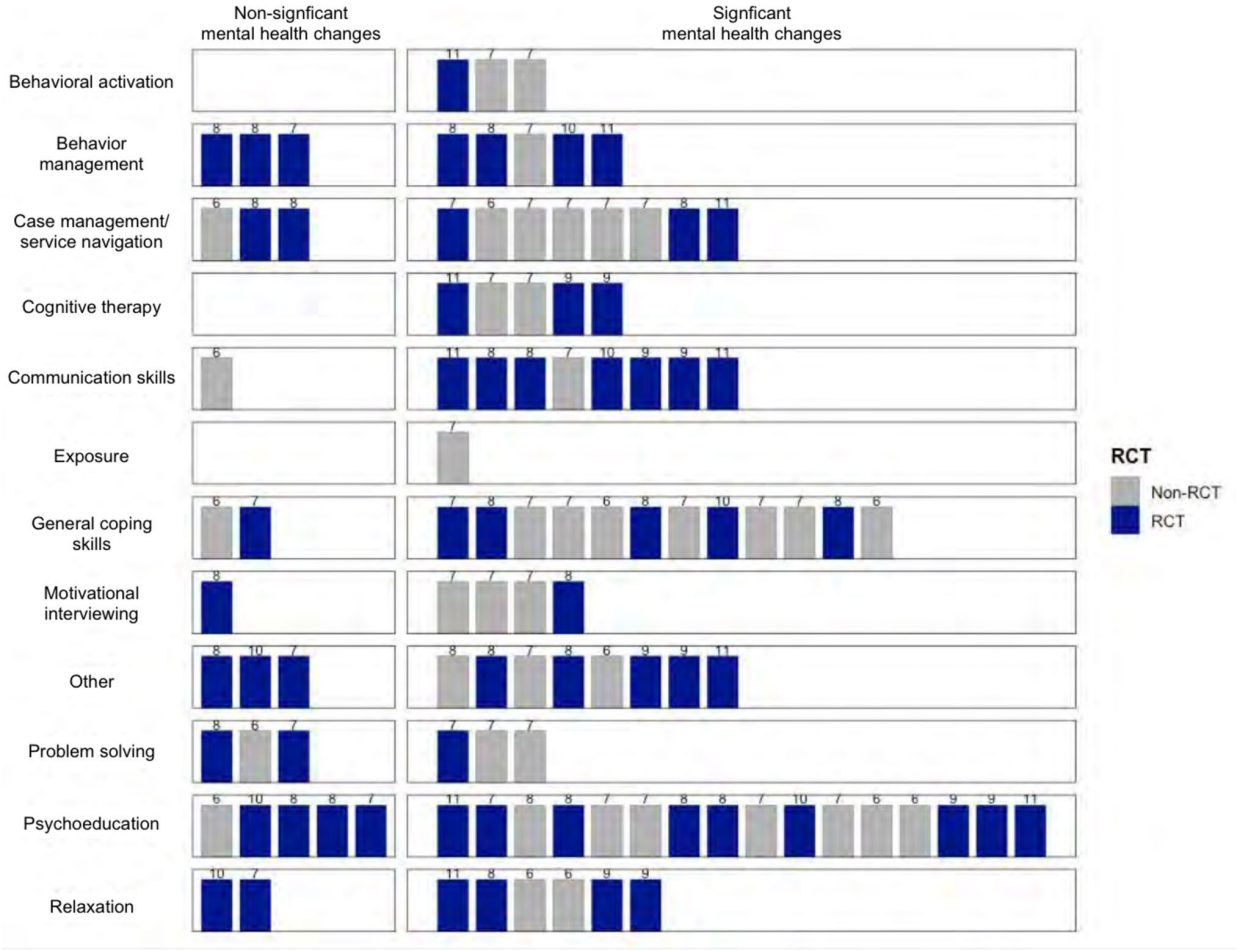
Harvest plot of outcomes across intervention components. Numbers above the bars denote the number of quality appraisal criteria met by a study. For RCTs, there was a total of 13 quality appraisal criteria. For non-RCTs, there was a total of 9 quality appraisal criteria

Table 4 summarizes the intervention outcomes and Table 5 summarizes effect size reporting across studies. Figure 2 presents a harvest plot illustrating outcomes across the different intervention components used in studies.

**Table 4:** Outcomes of CHW-delivered mental health interventions

Study	Significant improvement in mental health symptoms	High fidelity	High intervention participation	High satisfaction/acceptability
Alegría et al. (2019)	Y	Y	N	Y
Ashing and Rosales (2014)	Y	NR	Y	NR
Borelli et al. (2021)	Y	NR	NR	NR
Camacho et al. (2015)	Y	NR	N	NR
Conway et al. (2004)	N	NR	Y	NR
Crisanti et al. (2019)	Y	Y	N	NR
Cutshaw et al. (2012)	Y	NR	N	NR
Hernandez and Lucero (1996)	N	NR	Y	NR
Hovey et al. (2014)	Y	NR	Y	NR
Joachim-Célestin et al. (2022)	Y	NR	N	NR
Knox et al. (2011)	Y	Y	NR	Y
Lopez et al. (2019)	Y	NR	NR	NR
Magaña et al. (2015)	N	NR	NR	NR
Magaña et al. (2017)	Y	NR	Y	Y
Magaña et al. (2020)	Y	Y	Y	NR
Moore et al. (2016)	N	Y	Y	Y
Nicolaidis et al. (2013)	Y	NR	Y	Y
Norr et al. (2003)	N	NR	NR	NR
Rotheram-Fuller et al. (2017)	N	NR	NR	NR
Schepens Niemiec et al. (2018, 2021)	Y	Y	Y	Y
Schwingel et al. (2017)	Y	NR	N	Y
Spencer et al. (2013)	Y	NR	NR	NR
Tran et al. (2014)	Y	NR	NR	NR
Wagner et al. (2015, 2016)	Y	Y	N	Y
Williamson et al. (2014)	Y	NR	Y	NR

**Table 5:** Effect sizes

Study	Effect size reported	Effect size details
Alegría et al. (2019) †	Y	6mo follow-up Overall mood (HSCL-25): *d* =.27* Anxiety (GAD-7): *d* =.11 12mo follow-up Overall mood (HSCL-25): *d* =.24* Anxiety (GAD-7): *d* =.18
Ashing and Rosales (2014) †	N	
Borelli et al. (2021)	N	
Camacho et al. (2015)	N	
Conway et al. (2004) †	N	
Crisanti et al. (2019) †	N	
Cutshaw et al. (2012)	N	
Hernandez and Lucero (1996)	N	
Hovey et al. (2014)	Y	Post-treatment Depression (CES-D): *d* = 1.54* Migrant farmworkers stress (MFWSI): *d* =.64* Follow-up Depression (CES-D): *d* = 1.60* Migrant farmworkers stress (MFWSI): *d* =.72* Anxiety (PAI): *d* =.72 Hopelessness (BHS): *d* =.55 Self-esteem (Rosenberg Self-Esteem Inventory): *d* = 1.75
Joachim-Célestin et al. (2022)	N	
Knox et al. (2011) †	N	
Lopez et al. (2019) †	Y	Child outcomes Social communication (SCQ): *d* = 1.3* Maladaptive behaviors (SIB-R total): *d* =.8 Parent outcomes Autism parenting strategies efficacy: *d* =.8* Frequency of using autism parenting strategies: *d* = 1.2* Depression (CES-D): *d* =.7
Magaña et al. (2015) †	Y	Non-significant between-group differences in depression; both groups reported reductions in symptoms. Reported effect sizes for within-group pre/post differences: Intervention group pre/post Depression (CES-D): *d* =.4* Control group pre/post Depression (CES-D): *d* =.3*
Magaña et al. (2017)	Y	Caregiver outcomes Maternal autism knowledge questionnaire: *d* =.75* Autism parenting strategies efficacy: *d* = 1.17* Depression (CES-D): *d* = -.09 Strategy use frequency: *d* =.25 Child outcomes Language (ABC): *d* = -.61* Sensory (ABC): *d* =.08 Relating (ABC): *d* = -.20 Body use (ABC): *d* = -.19 Social & self-help (ABC): *d* = -.50 Internalizing (SIB-R): *d* =.21 Externalizing (SIB-R): *d* =.28 Asocial (SIB-R): *d* = -.25
Magaña et al. (2020) †	N	
Moore et al. (2016) †	Y	Non-significant between-group differences in alcohol intake; both groups improved. Reported effect sizes for non-significant differences: 6-week follow-up drinks per week:* d* =.77 12-week follow-up drinks per week: *d* =.41
Nicolaidis et al. (2013)	N	
Norr et al. (2003) †	N	
Rotheram-Fuller et al. (2017) †	N	
Schepens Niemiec et al. (2018, 2021)	Y	Pre/post-treatment Stress: *d* = -.39* Sleep disturbance (PSQI): *d* = -.30 Social activity satisfaction: *d* =.43 Pre-treatment/12-mo follow-up Stress: *d* = -.3 Sleep disturbance (PSQI): *d* = -.5* Social activity satisfaction: *d* =.5*
Schwingel et al. (2017)	N	
Spencer et al. (2013) †	Y	Diabetes-related emotional distress (PAID): *d* =.53* Depression (PHQ-2): *d* =.31* Note: Reporting"average intervention"effect sizes for the Latino sub-sample
Tran et al. (2014)	N	
Wagner et al. (2015, 2016) †	Y‡	Depression (PHQ-8): r-square =.086* Anxiety (PROMIS): r-square =.077* Diabetes distress (PAID): r-square =.000
Williamson et al. (2014) †	Y	Parenting skills: *d* =.60* Family cohesion: *d* =.31 Family support: *d* =.43* Family organization: *d* =.36* Child externalizing (SCBE-30): *d* =.09 Child internalizing (SCBE-30): *d* =.46*

Measure acronyms: HSCL-25: Hopkins Symptom Checklist-25; GAD-7: General Anxiety Disorder-7; CES-D: Center for Epidemiologic Studies Depression Scale; MFWSI: Migrant Farmworker Stress Inventory; PAI: Personality Assessment Inventory, Anxiety Scale; BHS: Beck Hopelessness Scale; SCQ: Social Communication Questionnaire; SIB-R: Scales of Independent Behavior, Revised; ABC: Autism Behavior Checklist; PSQI: Pittsburgh Sleep Quality Index; PHQ: Patient Health Questionnaire; PROMIS: APA Patient-Reported Outcomes Measurement Information System; PAID: Problem Areas in Diabetes; SCBE-30: Social Competence and Behavior Evaluation

^*^Significant effect sizes

^†^ RCTs

^‡^Effect sizes not reported in Wagner et al. (2015); only reported in the 2016 article

#### Mental Health Outcomes

Of the 25 studies included in this review, 19 (76%) reported improved mental health for individuals receiving the CHW-delivered intervention. Improved mental health outcomes were reported across all study designs, including 10 RCTs (71% of the RCTs in this review), nine single arm pre-post study designs (90% of pre-post studies), and one quasi-experimental study (100%). Ten studies (40%) reported the effect sizes associated with treatment outcomes. Significant Cohen’s d effect sizes ranged from an absolute value of 0.24 to 1.3. Significant changes in mental health included decreased depressive symptoms, anxiety symptoms, stress, hopelessness, distress, post-traumatic stress symptoms, sleep disturbance, number of “mentally unhealthy” days, and internalizing problems. Mental health improvements also included increases in parent–child attachment, parenting skills, family functioning, social problem-solving skills, and autism-related outcomes (e.g., social communication, language impairment). In addition to these mental health symptom outcomes, some studies also included broader wellbeing outcomes, reporting significant improvements in areas such as empathy, self-esteem, social support and satisfaction, and coping.

#### Dissemination & Implementation Science Outcomes

As an exploratory aim, we coded studies for the presence of any dissemination and implementation science (DIS) outcomes. DIS outcomes were informed by the RE-AIM (Reach, Effectiveness, Adoption, Implementation, Maintenance) Framework and the Implementation Outcomes Framework (IOF) (Glasgow et al., 2019; Proctor et al., 2011; Reilly et al., 2020; Reilly et al., 2020). Of the 25 studies included in this review, 17 studies (68%) reported some type of DIS outcome. DIS outcomes included 17 studies reporting participation, eight studies reporting satisfaction, and seven studies reporting fidelity. Of the 17 studies that reported participation rates, 10 studies (59%) reported high participation, defined as intervention completion rates of 75% or more. Across the eight studies that reported satisfaction, the metrics used for reporting satisfaction across studies varied, and included quantitative measures such as satisfaction and acceptance scales as well as qualitative reports of satisfaction from interviews and focus groups. While there was no consistent metric for reporting satisfaction across studies, all studies reported high levels of satisfaction based on authors’ quantitative or qualitative reports. Of the seven studies that reported fidelity, all studies reported high levels of fidelity, defined as > 75% fidelity as rated by each study’s respective fidelity scales.

### Quality Appraisals

For quality appraisal results we report counts based on the *27 articles* included in the review (rather than the *25 studies* represented by these 27 articles) given that each individual article was assessed for rigor.

#### RCTs

Fifteen articles were RCTs. Articles ranged from meeting 54–85% of the appraisal criteria (i.e., fulfilling 7–11 of the 13 criteria; see Table 6). The main contributing factors to risk of bias were unclear randomization procedures, lack of masking to treatment assignment, and inappropriate statistical analyses. Some studies stated only that participants were randomized, without reporting specific procedures for randomization. The lack of masking is understandable given the nature of these studies in which the control condition consisted of a waitlist, treatment as usual, or measurement only (no intervention). Hence, participants and interventionists (i.e., CHWs) would be aware of whether they were receiving/delivering a mental health intervention or not. Further, outcomes were often assessed via participant self-report measures, and since participants could not be masked to their condition, they also could not be masked assessors. Analytic shortcomings included an absence of reporting effect sizes or examination of attrition and low power.

**Table 6:** Quality appraisals for RCTs

	1. Was true randomiz-ation used for assignment of participants to treatment groups?	2. Was allocation to treatment groups conceal-ed?	3. Were treatment groups similar at the baseline?	4. Were participants blind to treatment assignment?	5. Were those delivering the treatment blind to treatment assignment?	6. Were treatment groups treated identically other than the intervention of interest?	7. Were outcome assessors blind to treatment assignment?	8. Were outcomes measured in the same way for treatment groups?	9. Were outcomes measured in a reliable way?	10. Was follow up complete and if not, were differences between groups in terms of their follow up adequately described and analysed?	11. Were participants analysed in the groups to which they were randomized?	12. Was appropriate statistical analysis used?	13. Was the trial design appropriate and any deviations from the standard RCT design accounted for in the conduct and analysis of the trial?
Alegría et al. (2019)	Yes	Yes	Yes	No	No	Yes	Yes	Yes	Yes	Yes	Yes	Yes	Yes
Ashing and Rosales (2014)	Yes	Yes	Yes	No	No	Yes	No	Yes	Yes	No	Yes	No	No
Conway et al. (2004)	Unclear	Unclear	Yes	No	No	Yes	Unclear	Yes	Yes	Yes	Yes	Yes	Yes
Crisanti et al. (2019)	Unclear	Unclear	Yes	No	No	Yes	Unclear	Yes	Yes	Yes	Yes	Yes	Yes
Knox et al. (2011)	Yes	Yes	Yes	No	No	Yes	No	Yes	Yes	No	Yes	No	Yes
Lopez et al. (2019)	Unclear	Unclear	Yes	No	No	Yes	Unclear	Yes	Yes	Yes	Yes	Yes	Yes
Magaña et al. (2015)	Yes	Yes	Yes	No	No	Yes	Unclear	Yes	Yes	Yes	Yes	Yes	Yes
Magaña et al. (2020)	Yes	Yes	Yes	No	No	Yes	Yes	Yes	Yes	Yes	Yes	No	Yes
Moore et al. (2016)	Yes	Yes	Yes	No	No	Yes	Unclear	Yes	Yes	No	Yes	No	Yes
Norr et al. (2003)	No	No	Yes	No	No	Yes	Yes	Yes	Yes	Yes	Yes	No	Yes
Rotheram-Fuller et al. (2017)	Unclear	Unclear	Yes	No	No	Yes	Unclear	Yes	Yes	No	Yes	Yes	Yes
Spencer et al. (2013)	Unclear	Unclear	Yes	No	No	Yes	No	Yes	Yes	Yes	Yes	Yes	Yes
Wagner et al. (2015)	Yes	Yes	Yes	No	No	Yes	No	Yes	Yes	No	Yes	Yes	Yes
Wagner et al. (2016)	Yes	Yes	Yes	No	No	Yes	Unclear	Yes	Yes	No	Yes	Yes	Yes
Williamson et al. (2014)	Yes	Yes	Yes	No	No	Yes	Yes	Yes	Yes	Yes	Yes	Yes	Yes

#### Quasi-Experimental/Pre-post

Twelve articles had a pre/post (n = 11) or quasi-experimental (n = 1) design. Articles ranged from meeting 67–89% of the appraisal criteria (i.e., fulfilling 6–8 of the 9 criteria). The primary drivers of risk of bias were inadequate analysis or reporting of attrition and inappropriate statistical analyses (see Table 7).

**Table 7:** Quality appraisals for quasi-experimental studies

	1. Is it clear in the study what is the ‘cause’ and what is the ‘effect’ (i.e. there is no confusion about which variable comes first)?	2. Were the participants included in any comparisons similar?	3. Were the participants included in any comparisons receiving similar treatment/care, other than the exposure or intervention of interest?	4. Was there a control group?	5. Were there multiple measurements of the outcome both pre and post the intervention/exposure?	6. Was follow up complete and if not, were differences between groups in terms of their follow up adequately described and analyzed?	7. Were the outcomes of participants included in any comparisons measured in the same way?	8. Were outcomes measured in a reliable way?	9. Was appropriate statistical analysis used?
Borelli et al. (2021)	Yes	Yes	Yes	No	Yes	Yes	Yes	Yes	Yes
Camacho et al. (2015)	Yes	Yes	Yes	No	Yes	Yes	Yes	Yes	No
Cutshaw et al. (2012)	Yes	Yes	Yes	No	Yes	Yes	Yes	Yes	No
Hernandez and Lucero (1996)	Yes	Yes	Yes	No	Yes	No	Yes	Yes	No
Hovey et al. (2014)	Yes	Yes	Yes	No	Yes	No	Yes	Yes	Yes
Joachim-Célestin et al. (2022)	Yes	No	Yes	No	Yes	No	Yes	Yes	Yes
Magaña et al. (2017)	Yes	Yes	Yes	No	Yes	No	Yes	Yes	Yes
Nicolaidis et al. (2013)	Yes	Yes	Yes	No	Yes	Yes	Yes	Yes	No
Schepens Niemiec et al. (2018)	Yes	Yes	Yes	No	Yes	Yes	Yes	Yes	No
Schepens Niemiec et al. (2021)	Yes	Yes	Yes	No	Yes	Yes	Yes	Yes	No
Schwingel et al. (2017)	Yes	Yes	Yes	No	Yes	No	Yes	Yes	No
Tran et al. (2014)	Yes	Yes	Yes	No	Yes	No	Yes	Yes	No

#### Qualitative

Six articles included qualitative data. Articles ranged from meeting 20−80% of the appraisal criteria (i.e., fulfilling 2–8 of the 10 criteria) (see Table 8). The main driver of risk of bias was a lack of reporting the specific orientation and/or qualitative methodology used. For example, studies stated that they conducted interviews or focus groups and analyzed the qualitative data, without specifying the philosophical perspective (e.g., pragmatist, constructivist, etc.) or the type of methodology (e.g., thematic analysis, content analysis, etc.), which are key components of rigorous qualitative and mixed methods research.

**Table 8:** Quality appraisals for qualitative portion of studies

	1. Is there congruity between the stated philosophical perspective and the research methodology?	2. Is there congruity between the research methodology and the research question or objectives?	3. Is there congruity between the research methodology and the methods used to collect data?	4. Is there congruity between the research methodology and the representation and analysis of data?	5. Is there congruity between the research methodology and the interpretation of results?	6. Is there a statement locating the researcher culturally or theoretically?	7. Is the influence of the researcher on the research, and vice- versa, addressed?	8. Are participants, and their voices, adequately represented?	9. Is the research ethical according to current criteria or, for recent studies, and is there evidence of ethical approval by an appropriate body?	10. Do the conclusions drawn in the research report flow from the analysis, or interpretation, of the data?
Knox et al. (2011)	No	No	No	No	No	No	No	Yes	No	Yes
Magaña et al. (2017)	No	No	No	No	No	No	No	Yes	Yes	Yes
Nicolaidis et al. (2013)	No	No	No	No	No	No	No	Yes	No	Yes
Schepens Niemiec et al. (2018)	No	No	No	No	No	No	No	Yes	Yes	Yes
Schepens Niemiec et al. (2021)	No	No	No	No	No	No	No	Yes	Yes	Yes
Schwingel et al. (2017)	Yes	Yes	Yes	Yes	Yes	No	No	Yes	Yes	Yes

## Discussion

This review aimed to synthesize the evidence for CHW mental health models for Latines in the US. Based on our review of 27 articles representing 25 studies, most of this research (76% of all studies, 71% of RCTs) found significant mental health symptom improvement associated with CHW-delivered interventions for Latine adults, seniors, and families. While these findings should be interpreted with caution, as there was a high risk of bias across studies, the reviewed literature provides valuable insights characterizing CHW models for Latines in the US and highlights areas for further research.

### Considerations for Intervention Content

The majority of studies found significant symptom reduction in mental health outcomes, which suggests that CHWs can effectively deliver interventions addressing mental health difficulties within the Latine community. Many of the delivered intervention elements aligned with evidence-based practices, such as cognitive therapy techniques (e.g., identifying thoughts, restructuring), relaxation and mindfulness practices, behavior management techniques (e.g., reinforcement, parent training), MI, and problem solving. However, there were also elements that were not well defined (i.e., reporting that an intervention addressed “stress management” or “coping skills” without providing a description of the technique or content). This points to a need for more clearly and consistently delineating the content of CHW mental health interventions in the literature.

Of the delivered intervention components, psychoeducation and coping skills were the most common, which aligns with the nature of the roles and tasks CHWs already engage in for any target health condition. Across their work in general health promotion, the CHW role often involves providing community members with information about health issues (e.g., psychoeducation) and supporting them in dealing with their health conditions (e.g., coping skills) (Scott et al., 2018). Intervention components identified in this review also mirror treatment elements common to CHW-delivered interventions across LMIC that are largely structured (i.e., have specific steps that CHWs can follow; see (Singla et al., 2017), which facilitates fidelity in implementation by non-specialists (e.g., Murray et al., 2014). As CHW mental health services are developed and implemented in the US, careful consideration will be necessary regarding what intervention components are included in these models to ensure feasibility and fidelity of implementation.

### Scope of Role for CHWs in Mental Health

Interventions in this review targeted a range of common mental health challenges, including depression, stress, parent–child relationships, substance use, and anxiety. Notably, while our search query had mesh terms that included serious mental illnesses (SMI) such as schizophrenia, bipolar, or personality disorders, none of the studies targeted SMI, suggesting that these mental health disorders may not be a good match for CHW delivered mental health interventions. While CHWs may be able to help support clients with SMI through some of the other common CHW role activities (e.g., outreach/navigation, auxiliary care; Barnett et al., 2018a, 2018b), for a direct interventionist role, CHWs seem best positioned to address common mental health challenges, whereas SMI may require a higher level of specialist care. Further, even within common mental health disorders, it is necessary to consider the level of severity to determine appropriateness for CHW-delivered intervention, as higher severity presentations would be beyond the scope of CHWs’ role (e.g., severe depression with suicidality). We argue that common mental health disorders of mild to moderate severity are likely most appropriate for CHW-delivered mental health interventions. However, this is an area in need of further research as studies to date have not examined symptom severity in relation to effectiveness and feasibility of CHW intervention. Additional research is also necessary to examine how the role of a more specialized co-provider (e.g., nurses, psychologists, social workers) could impact the feasibility of the types of mental health issues and severity levels that CHWs address as interventionists. In this review, only 4 studies had CHWs in a co-provider role, delivering intervention content alongside another provider, indicating a need for further research.

The fact that CHWs, a non-specialist workforce, are able to effectively deliver EBTs may raise a broader question: *can anyone deliver an EBT?* While the results of this review do not directly address this question, we posit several reasons for why CHWs are *uniquely* well positioned for EBT delivery. Reporting on CHW education and background was inconsistent across studies in this review. However, there are established national core competencies for CHWs which include skills in communication, interpersonal and relationship-building, education and facilitation, assessment, professional conduct, knowledge base, and advocacy, amongst others (Community Health Worker Core Consensus (C3) Project, 2018). The content of some of these core competencies overlap with basic clinical skills, such as active listening, communicating with empathy, documenting work, using MI skills, practicing cultural humility, and helping clients identify goals. In other words, CHWs are lay people from the community who *also* have key skills and competencies. It is the intersection of their lived expertise with these key competencies that, with appropriate training and supervision, make them uniquely positioned to engage clients in EBT delivery in ways that traditional licensed providers may not be able to. However, CHWs are not meant to be a replacement for licensed mental health providers and advanced mental health services. Rather, we promote the integration of CHWs in mental health services as an important *expansion* to the spectrum of care.

### Cultural Relevance of CHW Mental Health Models

Regarding cultural considerations for Latine populations, the majority of interventions were delivered to immigrant populations and were delivered in Spanish. Most studies reported some type of cultural tailoring, including incorporating culturally relevant values (e.g., *simpatia*, *personalismo*), topics or content (e.g., faith, acculturative stress), and delivery techniques (e.g., *dichos*, *cuentos*). Immigrant and non-English speaking populations are largely underserved in the US mental health service system due to a range of factors including structural barriers such as service accessibility, language barriers, cost and insurance coverage, as well as cultural factors such as stigma (Derr, 2016). It therefore follows that CHW mental health models are being leveraged to address treatment gaps for populations underserved in standard models of care given that CHWs can address some of their access barriers (i.e., language, stigma).

Having CHWs as a workforce with greater linguistic and cultural competencies than the existing mental health provider workforce, combined with cultural tailoring observed in these studies, is a particular strength and asset, as it well positions these models to better engage communities systemically excluded from traditional services. These CHW service models will become all the more necessary in the context of the US’ declining availability of Spanish-speaking mental health services (Pro et al., 2022), while, at the same time, the Spanish-speaking Latine population continues to grow. The majority of US children are from a minoritized racial or ethnic group, and immigrants are projected to be the primary driver of population growth (Vespa et al., 2020). Thus, the need for incorporating cultural tailoring approaches will continue to increase moving forward.

### Gaps in Understanding Implementation

The *who* and *how* of implementing CHW interventions serving U.S-based Latine populations are other domains in need of additional research. There was inconsistent reporting around the number of CHWs deployed in interventions, CHW demographics (i.e., gender, race, ethnicity, education), and CHW training and supervision. Given that articles in this review were outcomes-focused studies, it follows that process factors such as provider information, training and supervision were less commonly detailed. Yet, this information is critical for implementing and scaling CHW mental health models.

Peer-reviewed literature on the recruitment, training, and supervision of CHWs delivering mental health interventions is limited, and what is reported in the available literature provides a variable level of detail (Barnett et al., 2018a, 2018b; Schleiff et al., 2021; Singla et al., 2017). For example, one systematic review of CHW delivered mental health interventions found that the rationale for CHW selection was described by less than half of the studies (Singla et al., 2017), while another review noted that CHW selection criteria was not consistently described (Barnett et al., 2018a, 2018b). Reports of supervision practices are also variable, ranging from 55.6 to 64.1% of articles in systematic reviews reporting supervision information (Barnett et al., 2018a, 2018b; Singla et al., 2017). Training information is somewhat more commonly reported, but the primary content pertains to training duration (e.g., ranging from 2 days to 3 months [(Barnett et al., 2018a, 2018b)]; averaging 53.80 h [(Singla et al., 2017) and general training activities. The training and supervision findings from the present review mirror the gaps in the existing empirical evidence that reflect limited, variable, and surface-level reporting on these practices. Given this landscape, more recent writing on CHW training approaches has turned to case studies for guidance (Barnett et al., 2023; Schleiff et al., 2021). These case study analyses have highlighted the importance of active learning approaches for CHW training that provide opportunities for observation of the desired skills, including simulations, role plays, and supervised practice. In terms of content areas, trainings should supporting skill building in common factors (e.g., rapport building), intervention-specific techniques (e.g., behavioral activation), navigating challenging situations (e.g., conflict within families, client crises), as well as how to manage stress and burnout. The necessity of ongoing supervision, consultation, and monitoring has also been emphasized. For general CHW selection, training, and supervision practices, we refer readers to the World Health Organization’s guideline report, while keeping in mind that they caveat their guidelines with noting low evidence levels for most of the recommendations (World Health Organization, 2018).

Ultimately, further research is needed to empirically evaluate best practices for CHW selection, training, and supervision. For researchers, community practitioners, and service systems seeking to deploy CHW mental health interventions, it will be necessary to better understand questions such as: How should CHWs be selected for mental health interventionist roles? How should they be trained? What is a feasible caseload size? What type of supervision supports are needed? (i.e., supervision from whom? How often? What content should supervision cover?). Hence, in addition to a need for more rigorous outcome studies on CHW mental health models, there is a need for more detailed implementation reporting to inform domestic deployment of these services.

There are also additional implementation considerations at the sociopolitical level that, while not directly addressed by the studies in this review, are worth highlighting as they will impact the long-term sustainability and scalability of CHW mental health models for Latine and other communities. Namely, a key barrier to the widespread adoption of CHW mental health models is the absence of stable and dependable funding sources. Per a 2022 Medicaid report on CHW services, only one state included coverage of CHW counseling under their state Medicaid plan, and this was only for substance use screening and cessation (Medicaid and CHIP Payment and Access Commission, 2022). Therefore, federal and state-level policy changes are necessary to establish reliable funding mechanisms for these service models, thereby providing healthcare systems with a sustainable financial incentive to integrate CHWs into the mental health spectrum of care. Without such funding mechanisms, CHW mental health services will likely remain fragmented and limited in scope. Advancing these funding and policy changes will require advocacy on the part of researchers, CHWs and allied mental health professionals, as well as healthcare systems.

## Limitations

Several limitations are important to note. First, there was a high risk of bias across studies with regard to their methodological reporting and analyses. Shortcomings include failing to report randomization methods in some RCTs, absence of reporting effect sizes, inconsistent examination of attrition, lack of information on a priori power determination or low power, and inappropriate statistical analyses; studies with a qualitative component also often failed to report the specific orientation and/or the qualitative methodology used. As such, there is a need for more rigorous evaluations of CHW mental health models. Second, due to the nature of how results were reported across studies, including lack of effect sizes and inappropriate statistical analyses, we were not able to conduct a meta-analysis and instead used vote counting to summarize intervention effectiveness across this body of research (i.e., counting the number of studies with significant mental health effects, thereby giving equal weight to studies irrespective of sample size; Popay et al., 2006). Vote counting is useful in that it provides a broad characterization of possible effectiveness patterns, but it is an imprecise metric. Third, for DIS outcomes of satisfaction and fidelity, only a limited number of studies reported these outcomes (*n* = 8 and 7 respectively) and 100% reported high satisfaction and fidelity. There is the possibility of a reporting bias, such that studies with lower satisfaction or fidelity did not report this in their publications. Fourth, the scope of this review focused solely on CHWs in an interventionist role. There are a variety of other roles CHWs can occupy to support mental health (e.g., outreach and service navigation, auxiliary care and psychoeducation, etc.; Barnett et al., 2018a, 2018b). Their contributions to improving mental health through these other roles are not captured in this review. Lastly, there was limited research that could speak to the effectiveness of CHW-delivered mental health interventions as compared to those delivered by traditional mental health providers. Only one study in this review examined non-inferiority between CHW- and clinician-delivered intervention (Crisanti et al., 2019). This is a much needed area of further research to evaluate and benchmark the effectiveness of CHW services compared to existing models of mental healthcare.

## Conclusion

CHW-delivered mental health services for US Latines appear to be effective models for reducing the burden of common mental health disorders in this population, particularly for immigrant and Spanish-speaking Latines. CHW models that employ culturally-tailored and community-focused strategies may be more accessible and engaging than traditional mental health service models and thus expand reach and relevance for US Latines. Future directions for CHW intervention research should include more transparent reporting, with reduction in bias, on implementation processes (i.e., training, supervision, description of workforce and selection criteria), more robust statistical analyses, and comparison studies across provider groups such that we can more closely identify and scale out successful models into communities of need. Promising findings from studies to date indicate that additional, more rigorous research is warranted to better evaluate these models domestically.

## Supplementary Information

Below is the link to the electronic supplementary material.

Supplementary file1 (DOCX 27 KB)

## Footnotes

1 We use Latine as a pan-ethnic identifier for people of Latin American origin or descent. Further, we use Latine as a gender-inclusive term that facilitates integration and pronunciation in Spanish (Martínez & Gonzalez, 2021; Trejos, 2024)
