# Supplementary material for: Community Health Worker-Delivered Mental Health Interventions for Latine Populations in the U.S.: A Systematic Literature Review
Source: Adm Policy Ment Health. 2025 Jul 21;52(6):1174–98. doi: 10.1007/s10488-025-01459-6 (PMC12628377; doi:10.1007/s10488-025-01459-6)
Supplement: Supplementary file 1 — Supplementary file1 (DOCX 27 KB) [file 10488_2025_1459_MOESM1_ESM.docx]

**Appendix: Search Strategy**

A comprehensive literature search was developed and was run by an experienced medical librarian in June 2022 in the following databases: PubMed/MEDLINE, Scopus, CINAHL, and PsycINFO. Google Scholar was searched as well. Both controlled vocabularies (e.g. MeSH terms) and keywords in the title or abstract fields were searched. There were no restrictions on geography, age of participants or language of publication. Additionally, a hand search was conducted of the reference lists of selected articles. A reproducible search strategy is below.

PubMed:

("community health"[Title/Abstract] OR "family support"[Title/Abstract] OR lay[Title/Abstract] OR natural helper*[Title/Abstract] OR outreach[Title/Abstract] OR paraprofessional*[Title/Abstract] OR peer*[Title/Abstract] OR patient navigator*[Title/Abstract] OR promotora*[Title/Abstract])

AND (worker*[Title/Abstract] OR aide*[Title/Abstract] OR advisor*[Title/Abstract] OR advocate*[Title/Abstract] OR coach*[Title/Abstract] OR coordinator*[Title/Abstract] OR counselor*[Title/Abstract] OR educator*[Title/Abstract] OR employee*[Title/Abstract] OR liaison*[Title/Abstract] OR mentor*[Title/Abstract] OR navigator*[Title/Abstract] OR natural helper*[Title/Abstract] OR paraprofessional*[Title/Abstract] OR patient navigator*[Title/Abstract] OR promotora*[Title/Abstract] OR provider*[Title/Abstract] OR promoter*[Title/Abstract] OR representative*[Title/Abstract] OR specialist*[Title/Abstract])

AND (mental health[Title/Abstract] OR alcohol abuse[Title/Abstract] OR alcohol use[Title/Abstract] OR heavy drinking[Title/Abstract] OR anxiety[Title/Abstract] OR autis*[Title/Abstract] OR asperger*[Title/Abstract] OR behavioral health[Title/Abstract] OR behavioural health[Title/Abstract] OR conduct disorder[Title/Abstract] OR depression[Title/Abstract] OR disruptive behavior[Title/Abstract] OR intellectual disabilit*[Title/Abstract] OR intellectual and developmental disabilit*[Title/Abstract] OR IDD[Title/Abstract] OR opioid use[Title/Abstract] OR parenting[Title/Abstract] OR parent training[Title/Abstract] OR parent management training[Title/Abstract] OR PMT[Title/Abstract] OR PTSD[Title/Abstract] OR post traumatic stress disorder[Title/Abstract] OR psychosis[Title/Abstract] OR stress[Title/Abstract] OR substance abuse[Title/Abstract] OR substance use[Title/Abstract] OR trauma[Title/Abstract] OR "Intellectual Disability"[Mesh] OR "Community Mental Health Services"[Mesh] OR "Mental Disorders"[Mesh] OR "Behavioral Symptoms"[Mesh] OR "Mental Health Services"[Mesh])

AND (Latinx[Title/Abstract] OR Latino*[Title/Abstract] OR Latina*[Title/Abstract] OR Cuban[Title/Abstract] OR Chicana[Title/Abstract] OR Chicanas[Title/Abstract] OR Chicano[Title/Abstract] OR Chicanos[Title/Abstract] OR Hispanic[Title/Abstract] OR Mexican American[Title/Abstract] OR Puerto Rican[Title/Abstract] OR promotora*[Title/Abstract] OR underserved[Title/Abstract] OR medicaid[Title/Abstract] OR Latino[Mesh] OR "Mexican Americans"[Mesh])
